# Characterization of Glioblastoma Cells Response to Regorafenib

**DOI:** 10.3390/cancers14246193

**Published:** 2022-12-15

**Authors:** Maria Patrizia Mongiardi, Mariachiara Buccarelli, Alessia Formato, Elisa Orecchini, Maria Salbini, Valentina Ricci, Tiziana Orsini, Sabrina Putti, Silvia Chiesa, Lucia Ricci-Vitiani, Quintino Giorgio D’Alessandris, Roberto Pallini, Andrea Levi, Maria Laura Falchetti

**Affiliations:** 1 Institute of Biochemistry and Cell Biology, IBBC-CNR, Campus Adriano Buzzati Traverso, Via Ercole Ramarini 32, Monterotondo Scalo, 00015 Rome, Italy; 2 Department of Oncology and Molecular Medicine, Istituto Superiore di Sanità, Viale Regina Elena 299, 00161 Rome, Italy; 3 Institute of Neurosurgery, Catholic University School of Medicine, L.go F. Vito 1, 00168 Rome, Italy; 4 Department of Radiation Oncology, Fondazione Policlinico Universitario A. Gemelli IRCCS, Università Cattolica del S. Cuore, L.go A. Gemelli, 8, 00168 Rome, Italy; 5 Institute of Neurosurgery, Fondazione IRCCS Policlinico Universitario A. Gemelli, 00168 Rome, Italy

**Keywords:** glioblastoma, therapy, regorafenib, glioma stem cells (GSCs), epithelial to mesenchymal transition (EMT)

## Abstract

**Simple Summary:**

Glioblastoma is the most aggressive primary brain tumor, characterized by a short survival and by an invariably poor outcome. The clinical management of glioblastoma patients is based on surgery followed by adjuvant radio-chemotherapy. Glioblastoma therapy remained substantially unaltered in the last two decades, due to the lack of significant therapeutic alternatives. Regorafenib, a multikinase inhibitor already used as an anticancer drug for hepatocellular carcinoma, has recently been introduced as a therapy for relapsed glioblastoma, based on the encouraging results of a randomized phase II clinical trial. However, very little is known about the mechanisms governing glioblastoma cells’ response to regorafenib. Here we present an in vitro study, performed on glioblastoma tumor cells and on patient-derived glioma stem cells, aiming at characterizing the cellular response to regorafenib. Overall, the emerging message is that regorafenib limits glioblastoma cell proliferation, but might eventually increase the tumor cells’ migration ability.

**Abstract:**

Glioblastoma (GBM), the most malignant primary brain tumor in adults. Although not frequent, it has a relevant social impact because the peak incidence coincides with the age of professional maturity. A number of novel treatments have been proposed, yet clinical trials have been disappointing. Recently, a phase II clinical trial (REGOMA) demonstrated that the multikinase inhibitor regorafenib significantly increased the median overall survival (OS) of GBM patients when compared to lomustine-treated patients. On this basis, the National Comprehensive Cancer Network (NCCN) 2020 Guidelines included regorafenib as a preferred regimen in relapsed GBM treatment. Despite the use in GBM patients’ therapy, little is known about the molecular mechanisms governing regorafenib effectiveness on the GBM tumor. Here we report an in vitro characterization of GBM tumor cells’ response to regorafenib, performed both on cell lines and on patient-derived glioma stem cells (GSCs). Overall, regorafenib significantly reduced cell growth of 2D tumor cell cultures and of 3D tumor spheroids. Strikingly, this effect was accompanied by transcriptional regulation of epithelial to mesenchymal transition (EMT) genes and by an increased ability of surviving tumor cells to invade the surrounding matrix. Taken together, our data suggest that regorafenib limits cell growth, however, it might induce an invasive phenotype.

## 1. Introduction

Glioblastoma (GBM) is the most malignant and, unfortunately, the most frequent primary brain tumor in adults. It is a highly vascularized cancer whereby neo-angiogenesis is one hallmark for histological diagnosis. Standard-of-care treatment consists of surgery followed by chemo-radiation. Nevertheless, median survival is 14.6 months [1]. This is mainly due to the tendency of the tumor to give recurrences, which can be close to the initial site, in a distant site, multifocal or diffusely infiltrating. A number of novel treatments have been proposed based on therapeutic efficacy in vitro and in various models, yet clinical trials have been disappointing. Failures are due to several factors including (i) inter- and intra-individual heterogeneity, whereby in the very same GBM coexist multiple cell populations with different driver oncogenic pathways; (ii) inadequate tumor modeling; (iii) presence of glioma stem-like cells (GSCs) resistant to chemotherapy and radiation [2,3]; (iv) highly invasive nature causing infiltrative cells to persist after surgery. GSC-based research has the potential to overcome these drawbacks. In fact, GSCs derived from surgical specimens closely mimic the molecular profile of the GBM patient, reproduce the infiltrative behavior when implanted in vivo, and generate a heterogeneous cellular progeny. Recent advances in the understanding of the molecular basis of gliomagenesis have led to the development of therapies which target tumor angiogenesis via inhibition of vascular endothelial growth factor (VEGF) [4] and its receptors (VEGFRs) [5,6]. Unfortunately, antiangiogenic therapies have substantially disappointed the expectations, probably due to tumor escape mechanisms.

Multikinase inhibitors, molecules targeting a panel of different kinases, have been evaluated in several studies for recurrent GBM therapy [7,8] with the aim of targeting different tumor-related pathways, including invasion and metastasis, cell growth and survival, and neo-angiogenesis. Among multikinase inhibitors, regorafenib, a sorafenib derivative, already in clinical use for metastatic colorectal cancers and hepatocellular carcinomas, recently showed interesting results in a phase II clinical trial on recurrent GBM (REGOMA) [9]. Regorafenib targets angiogenic kinases (VEGFR 1-3, PDGFR-b) as well as oncogenic kinases (c-KIT, RET, FGFR, Raf). Besides targeting GBM cells, the main therapeutic effects of regorafenib are anti-angiogenesis and the remodeling of the tumor microenvironment through several mechanisms of action. A REGOMA trial was performed on a cohort of 119 relapsed GBM patients. The study showed a longer overall survival (OS) (7.4 months compared to 5.6 months with lomustine), and a statistically significant improvement of 6-month progression free survival (PFS) in the regorafenib arm when compared to the control lomustine-treated group. Some concerns on the REGOMA trial design have been formulated, particularly regarding the poor outcome of the control arm compared with other studies, the presence of IDH-mutated patients in the regorafenib arm, and the lack of centralized pathology and molecular review [10]. This notwithstanding, regorafenib is considered as the first drug, since the introduction of temozolomide, about 20 years ago, to demonstrate efficacy in GBM treatment. On this basis, the National Comprehensive Cancer Network (NCCN) 2020 Guidelines included regorafenib as a preferred regimen in relapsed GBM treatment and the Italian Agency of Medicine (AIFA) approved regorafenib for Italian recurrent GBM patients.

A very recent phase II study, analyzing patient-reported outcomes of regorafenib compared with lomustine, revealed that regorafenib did not negatively affect the health-related quality of life [11], further supporting the clinical use of the drug. Despite regorafenib entering clinical practice for the treatment of relapsed GBM, the molecular mechanisms governing GBM patients’ sensitivity to regorafenib are still poorly understood. The REGOMA team reported a first retrospective analysis on patients enrolled in the trial, in which transcriptional profiling of tumor specimens revealed a mini signature correlating with increased OS [12]. This mini signature is based on the expression of five biomarkers: elevated expression of HIF1A and CDKN1A mRNA, and reduced expression of miR-93-5p, miR3607-3p, and miR-301a-3p, as obtained analyzing RNA from tumor samples of 72 patients (36 in the regorafenib and 36 in the lomustine arm). In another paper from the same group, activation of the liver kinase B1/AMP kinase (AMPK) pathway, as shown by increased immunohistochemical expression of phosphorylated acetyl-CoA carboxylase (pACC), was correlated with improved response to regorafenib [13]. Recently, Jiang and colleagues demonstrated that regorafenib induces autophagic arrest of GBM cells [14], providing a key to reading regorafenib-dependent anti-tumor activity.

Despite the encouraging results from the REGOMA trial, the real benefit of regorafenib use in the clinical management of relapsed GBM remains to be fully addressed. Although it is undoubted that there is a subgroup of patients who respond to regorafenib [9,15], the drug effectiveness is not ubiquitous and a more comprehensive understanding of molecular determinants of regorafenib responsiveness across this heterogeneous cancer is mandatory.

Here we present an in vitro characterization of GBM cells’ response to regorafenib, using both tumor cell lines and GSCs. Our study shows that, in vitro, regorafenib limits tumor cells viability but promotes epithelial to mesenchymal transition (EMT) and tumor cells’ migratory ability.

## 2. Materials and Methods

### 2.1. Cell Culture

U87 (alias U87-MG) and A172 were purchased from ATCC and cultured in DMEM 4.5 g/L glucose (Thermo Fisher Scientific, Waltham, MA, USA), supplemented with 10% fetal bovine serum (Thermo Fisher Scientific). GSCs were isolated from patients’ surgical samples at the Institute of Neurosurgery, Catholic University of Rome as previously described [16] and grown in suspension in DMEM/F12 serum-free medium (Thermo Fisher Scientific) containing 2 mM L-glutamine, 0.6% glucose, 9.6 mg/mL putrescine, 6.3 ng/mL progesterone, 5.2 ng/mL sodium selenite, 0.025 mg/mL insulin, 0.1 mg/mL transferrin sodium salt (Sigma Aldrich, St Louis, MO, USA), EGF (20 ng/mL; Peprotech, London, UK), bFGF (10 ng/mL; Peprotech), and heparin (2 μg/mL; Sigma Aldrich) at 37 °C, 5% CO_2_. All cell lines were regularly checked to exclude mycoplasma contamination by Mycoalert Detection Kit (Lonza, Basel, Switzerland).

Regorafenib (Bay73-4506) was purchased from Selleckem (Houston, TX, USA) and resuspended in DMSO.

### 2.2. Viability Assay

The effect of regorafenib exposure on cell viability was evaluated by the Cell Titer 96 Aqueous One Solution Cell Proliferation Assay (Promega, Madison, WI, USA) or by CellTiter-Glo™ (Promega), according to the manufacturer’s instructions. Briefly, U87 and A172 were seeded in technical triplicate on a 96-well microplate. Twenty-four hours post-plating, we added regorafenib. MTS was performed 48 and 72 h later. For GSCs, cells were dissociated and seeded in technical triplicate on a 96-well microplate. Twenty-four hours post-plating, we added regorafenib. ATP levels were measured 48 and 72 h later using the CellTiter-Glo™. The mean of the raw luminescence values (LD) from triplicate wells treated with vehicle alone (DMSO 0.2%, mLC), was used as reference to calculate percent viability from wells treated with drugs (VD), using the following formula: VD = (LD/mLC) × 100, as previously described [17].

### 2.3. IC50 Calculation

IC50 was calculated for U87, A172, GSC#1, GSC#83, GSC#61, GSC#366, GSC#450 and GSC# 493 by GraphPad Prism (GraphPad Prism version 8.0 for Windows, GraphPad Software, San Diego, CA, USA). Briefly, the dose-response curve to regorafenib obtained as reported in the *Viability assay* paragraph was fitted in Prism to determine the IC50, at 48 (U87 and A172) and 72 h (GSCs) of regorafenib exposure, specific for each cell strain.

### 2.4. Cell-Cycle Analysis

Cell-cycle analysis was performed as in [18]. Briefly, U87, A172, GSC#1, and GSC#83, were treated in biological triplicate with regorafenib and harvested at the indicated time points. Cells were fixed in 70% ice-cold ethanol for at least 18 h and treated with RNase A (100 µg/mL; Promega) for 15 min at 37 °C. Cells were stained with propidium iodide (100 µg/mL; Thermo Fisher Scientific) for 20 min at 37 °C and sorted on a fluorescence-activated cell sorter FACS CANTO II (Becton Dickinson BD, Franklin Lakes, NJ, USA). Cell populations were selected by SSC and FSC parameters and were excluded doublets. The results were analyzed with FlowJo software (BD).

### 2.5. Annexin V Staining

U87, A172, GSC#1, and GSC#83 were treated with regorafenib (7.5 µM) for 24 h and 48 h. Apoptosis was analyzed using flow cytometry (FACSCantoII flow cytometer, BD Biosciences, Franklin Lakes, NJ, USA). Cells were stained with annexin V-FITC (BD Biosciences) and propidium iodide (PI) according to the manufacturer’s instructions.

The experiment was performed in triplicate, and the proportions of apoptotic cells were compared between the regorafenib-treated and untreated groups, using FlowJo (ver.10.8) (BD Biosciences).

### 2.6. Clonogenesis Assay

Colony-formation ability of GSCs after regorafenib treatment was evaluated by plating a single cell/well in 96-well plates. The experiments were performed in GSC complete medium in the presence of regorafenib or DMSO as control. He dose of regorafenib was 2.5 µM, and the drug was added again after 1 week. After 2 weeks, each well was examined and the number of spheres/cell aggregates was counted.

Results are shown as percent colony number values from independent experiments in triplicate, calculated over the correspondent control and are shown as mean ± SD. **, *p* < 0.01; ***; *p* < 0.001 based on Student’s *t* test.

### 2.7. Reverse Transcription and Real Time PCR Analyses

One μg of RNA isolated by TRIzol (Thermo Fisher Scientific) was retrotranscribed with MLV-Reverse Transcriptase (Promega) and amplified by real-time PCR using SYBR Select Master Mix (Applied Biosystem, Foster City, CA, USA) and gene specific-primers (Table 1). Real-time PCR was performed with the 7900HT Fast Real-Time PCR System by Applied Biosystem. Statistical analysis was performed using Prism software (GraphPad software). Mean values and standard deviation were generated from at least three biological replicates.

### 2.8. Western Blot Analysis

U87, A172, GSC#1, and GSC#83 were treated with 7.5 µM regorafenib for 48 and 72 h. Protein extraction was performed using protein buffer extraction (50 mM Tris-HCl pH 7.5, 150 mM NaCl, 5 mM EDTA, 5 mM EGTA pH 8.1% NP40) supplemented with protease and phosphatase inhibitors. Protein concentration was determined using Bradford assay (BioRad, Herculec, CA, USA) and 15 µg of protein were resolved by SDS-PAGE, transferred onto nitrocellulose membrane, and then analyzed by immunoblotting using specific antibodies: p-p53 ser15 (Cell Signaling 9284, Danvers, MA, USA) 1:1000; p53 (Santa Cruz sc-6243, Dallas, TX, USA) 1:500; Actin (Invitrogen Ma1-744, Waltham, MA, USA) 1:5000; AKT (Cell Signaling 9272) 1:1000; and p-akt ser473 (Cell Signaling 4058) 1:1000; p21 (Cell Signaling 2947) 1:1000. Graphs report quantitation of the proteins levels as addressed by Image Lab Software (BioRad).

### 2.9. Tumor Spheroid Three-Dimensional Invasion Assay

Cells were suspended in 20% methylcellulose in complete growth medium (2 × 10^3^ cells/spheroid in 100 µL) and plated on ultra-low attachment (ULA) 96-well plates (Corning, Corning, NY, USA). After 48 h, cellular media and regorafenib or vehicle were added. Media were changed every three days. For collagen-embedded spheroids, 2 × 10^3^ A172 or 10^3^ U87, GSC#1, and GSC#83 were suspended in 20% methylcellulose solution and plated on ULA plates. After 48 h, the spheroids were embedded into collagen matrix as indicated in [19]. Spheroid area, diameters, and migration distance were measured by ImageJ software. In particular, for migration distance we arbitrarily set four cardinal points per spheroid. For each point, we measured the distance between the edge of the spheroid and the furthest cell and calculated the mean value from the four measurements per spheroid. The mean and standard deviation of at least five spheroids are shown.

### 2.10. Fluorescent Staining Protocol

Tumor spheroids, obtained as previously described, were incubated for one hour with propidium iodide (4 µg/mL; Thermo Fisher Scientific), calcein AM (1 µM; Sigma Aldrich), and Hoechst (10 µg/mL; Sigma Aldrich) in culture medium. After incubation, the medium was removed and replaced by PBS solution. Images were acquired by laser confocal microscope (Olympus FV1200, Tokyo, Japan).

### 2.11. X-ray Microtomography (microCT)

This technique allows the fine analysis of spheroid volumes, being a bioimaging system that develops a three-dimensional virtual model of the sample in a non-destructive way and with a micrometric spatial resolution. Samples were fixed in 3.7% formaldehyde for 15 min, then treated with potassium iodine contrast agent, 0.1N (*v*/*v*) Lugol solution (Sigma Aldrich) for 72 h at room temperature. Spheroids were individually transferred and analyzed in 0.5 mL microtubes. The acquisition of the 3D datasets was performed using a high-resolution 3D Micro-CT Imaging System (Skyscan 1172G Bruker, Kontich, Belgium), with a camera pixel/size of 4.8 µm, tube voltage peak of 39 kV, exposure time of 170 ms, with no physical filtration. Captured data were reconstructed using built-in NRecon Skyscan Bruker Software (Version:1.6.6.0) and processed with CTvox 3D-Visualization software (v. 2.5, Bruker) to create 3D images; DataViewer v. 1.4.4 (Skyscan Bruker software) was used to generate volume rendering and virtual sectioning views [20,21].

### 2.12. Statistical Analysis

Data are expressed as mean ± SD as indicated in figure legends. Mean values and standard deviation were generated from at least three biological replicates. Significance was calculated using a two-tailed t test and *p* value of <0.05 were considered significant. Statistical analysis was performed using Prism software (GraphPad software).

## 3. Results

### 3.1. Cell Viability, Cell-Cycle Regulation, and Neurosphere-Forming Ability upon Regorafenib Exposure

**Figure 1:**
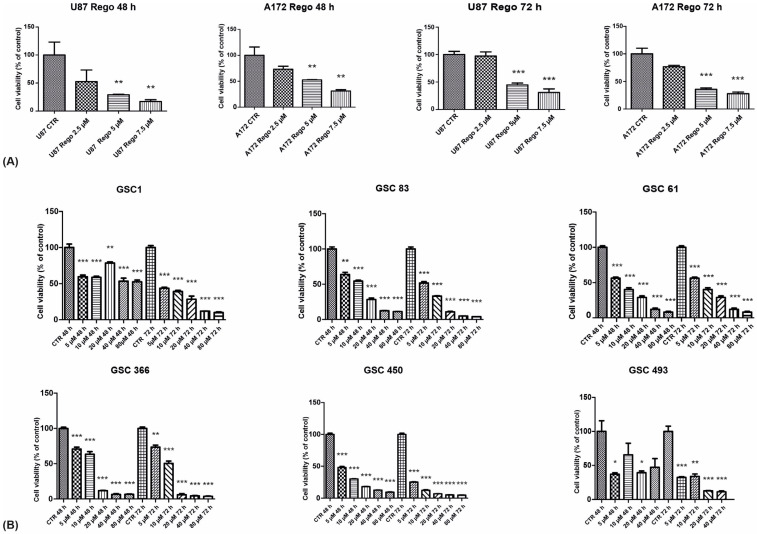
Cell viability assay of regorafenib-treated cells. U87 and A172 cells were vehicle-treated (CTR) or treated with regorafenib concentrations in the range 2.5–7.5 µM for 48 and 72 h before cell viability assay (**A**). Patient-derived GSCs were treated with regorafenib concentrations in the range 5–40 µM for 48 and 72 h before cell viability assay (**B**). *n* = 3 biological replicates. * *p* value < 0.05; ** *p* value < 0.01; *** *p* value < 0.001.

As a first step in characterizing GBM cells’ response to regorafenib, we evaluated cell viability by MTS assays on two GBM cell lines, U87 and A172, and on six patient-derived GSCs established in our laboratory (GSC#1, GSC#61, GSC#83, GSC#366, GSC#450, and GSC#493) (Table 2). U87 and A172 are widely accepted cellular models of GBM tumor cells [22,23]. Both of them are able to grow as 3D cellular cultures, while only U87 develop brain tumors upon grafting in animal models. GSCs, as reported above, represent the cellular subpopulation responsible for drug resistance. The rationale for testing regorafenib sensitivity on a wide range of GSC strains stems from their well-established role in drug resistance [24,25]. Since it is known that GSCs are more resistant to anticancer drugs than tumor cell lines (for a recent review see [26]), we assayed a higher drug concentration range in GSCs than in U87 and A172. Based on literature data [14], we treated cells with regorafenib concentrations ranging from 0.5 to 7.5 µM and from 5 to 40 µM in established cell lines and in GSCs, respectively. Figure 1 shows the cell viability curves obtained. U87 and A172 exhibited similar sensitivity to regorafenib, with a peak of viability reduction at 48 h of treatment (Figure 1A). Conversely, variable sensitivity to regorafenib was observed in the patient-derived GSCs (Figure 1B). The kinetics of response to regorafenib in GSCs was similar although slightly delayed with respect to U87 and A172. GSCs exhibited a more pronounced reduction of cell viability after 72 h rather than after 48 h exposure. Table 3 shows the IC50 values calculated for U87 and A172 and for all the six GSC strains tested.

To characterize regorafenib impact on cell-cycle regulation, we analyzed, by FACS, propidium iodide-labeled U87 and A172 cells. We extended the analysis to two GSC lines, GSC#1 and GSC#83. We selected these two GSC lines because they are the better characterized among our collection [16,27] and because of their biological and metabolic characteristics. Although they both derived from tumors localized close to the temporal subventricular zone, the patients from which they were raised have different OS (12.5 and 8.5 months for line #1 and line #83, respectively), different MGMT status (methylated for line #1, unmethylated for line #83), different CD133 positivity (more than 80% in line #1 and less than 1% in line #83), and grow as neurospheres (line #1) or mostly as a monolayer (line #83). Further, GSC#1 and GSC#83 belong to different metabolic and genetic clusters [16,27,28,29].

**Figure 2:**
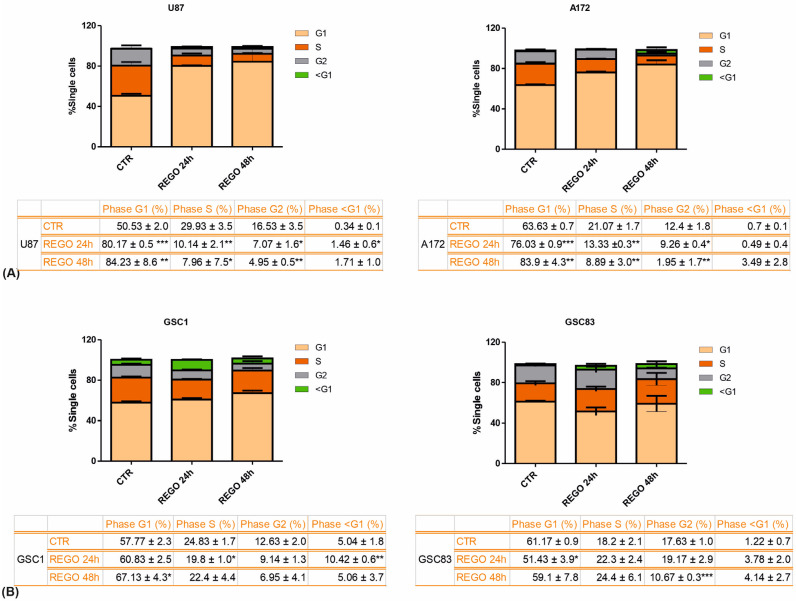
FACS analyses of cell cycle upon regorafenib treatment. Established cell lines (**A**) and patient-derived GSCs (**B**) were vehicle-treated (CTR) or treated with 7.5 µM regorafenib for 24 or 48 h before fixation, propidium iodide staining, and FACS analysis. *n* = 3 biological replicates. * *p* value < 0.05; ** *p* value < 0.01; *** *p* value < 0.001.

Cells were exposed to 7.5 µM regorafenib for 24 or 48 h before propidium iodide labeling and FACS analysis (Figure 2 and Figure S1). Overall, U87 and A172 exhibited a block in G1, clearly detectable at 24 h of regorafenib exposure, with a concomitant reduction of the percentage of cells in S and G2. The cell-cycle distribution of GSCs exposed to regorafenib differs from the one observed in U87 and A172, with the most apparent difference being an increase in the subG1 phase. Moreover, the percentages of cells in G1 and S seem not to be significantly affected by regorafenib. This different behavior between established cell lines and GSCs is not surprising, since it has been previously reported when comparing GSCs and GBM cell lines’ response to radiation [30].

Finally, we addressed the impact of regorafenib on the ability of GSCs to form spheres. It has been previously reported that regorafenib impairs this ability [31], which is an indicator of the cells self-renewal capability, a major stem-cell feature. To address this specific issue in our experimental model, we performed a clonogenic assay. Briefly, GSC#1 and GSC#83 were seeded at a density of a single cell/well in 96-well plates. After two weeks of regorafenib exposure, the number of spheres/cell aggregates were counted. Supplementary Figure S2 shows that regorafenib significantly impairs the GSCs ability to develop spheres, consistent with literature data.

### 3.2. Evaluation of Apoptosis in Regorafenib-Treated Cells

**Figure 3:**
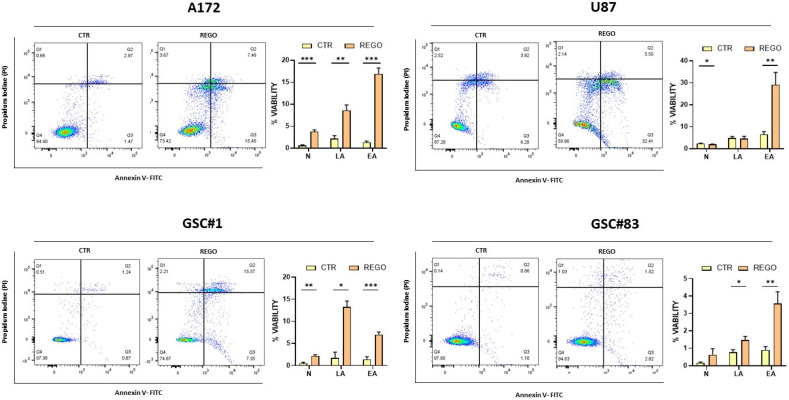
Annexin staining of cells treated with regorafenib for 48 h. Cells were double stained by annexin V and by propidium iodide, for apoptotic and dead-cell labeling, respectively. N: necrotic cells; LA: late apoptosis; EA: early apoptosis. *n* = 3 biological replicates. * *p* value < 0.05; ** *p* value < 0.01; *** *p* value < 0.001.

We then wondered if regorafenib negatively affects GBM cells viability through induction of apoptosis. To address this point, we performed fluorescent annexin V/propidium iodide (PI) double-staining assay on U87, A172, GSC#1, and GSC#83 treated with regorafenib for 24 (Supplementary Figure S3) or 48 h (Figure 3). Although regorafenib induces cell death, apoptosis does not seem to represent the principal mechanism through which regorafenib works in our cellular models. U87 and A172 show an increase in the percentage of apoptotic cells following regorafenib exposure which, although modest, is more pronounced than the one exhibited by GSCs. Notably, in accordance with cell viability assays (Figure 1), annexin V staining reveals a delayed kinetic in the response to regorafenib in GSC cells when compared to U87 and A172. We can speculate that regorafenib might induce cell death according to different routes, i.e., through autophagy, as recently demonstrated [14], with apoptosis playing a minor role.

**Figure 4:**
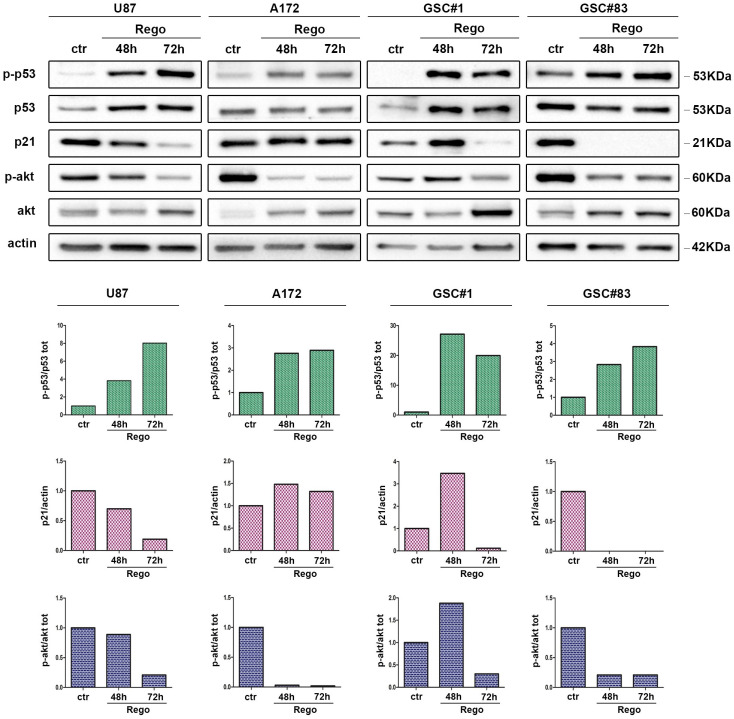
Protein expression of apoptosis-related proteins in regorafenib-treated GBM cells. U87, A172, GSC#1, and GSC#83 were treated with regorafenib for 48 and 72 h. Figure shows a representative Western Blot analysis of total and phosphorylated p53 protein, of p21, and of phosphorylated AKT, analyzed as an indirect strategy to confirm regorafenib effectiveness in our cells. Actin was used as protein-loading control (Original western blot can be found in Supplementary Figure S4). Graphs report quantitation of the proteins levels as addressed by Image Lab Software. Experiments were repeated three times.

To further explore the mechanisms at the basis of regorafenib-induced cell death in our GBM cell models, we examined, by Western blot, the expression of p53 and p21, two proteins fundamental in regulating the cellular apoptotic response. Finally, we looked at AKT phosphorylation as an indirect strategy to confirm regorafenib effectiveness in our cellular models. AKT signaling pathway is indeed common to several tyrosine kinases targeted by regorafenib. Figure 4 shows that either GBM cell lines or GSCs experience a consistent up-regulation of total and phosphorylated p53 protein. Notably, p21 was down-regulated upon regorafenib treatment. This inverse relationship between p53 and p21 expression was quite unexpected, since it in some way contradicts the classical idea of p21 up-regulation as a consequence of p53 increase (i.e., upon DNA damage). Interestingly, in our experimental models, p21 protein expression parallels that of pAKT, with the exception of the A172 cell line, where, despite a considerable decrease of pAKT, we observe an increase of p21 protein expression. Since AKT phosphorylation of p21 has already been observed [32,33,34], we hypothesize that regorafenib-dependent dephosphorylation of AKT results in a decreased expression of p21.

### 3.3. Transcriptional Regulation of Genes of Epithelial to Mesenchymal Transition (EMT) in Regorafenib-Treated Cells

Regorafenib targets different pathways, including the pathway of VEGF, which is particularly relevant in GBM growth. Previous attempts to restrain GBM growth targeting VEGF or its receptors, either by bevacizumab (a humanized monoclonal antibody specific for VEGF), by VEGF-trap (a soluble decoy receptor for VEGF) or by anti-VEGFR antibody, resulted in the improvement of tumor cells’ ability to invade brain parenchyma, a phenotypic change known as infiltrative shift [35,36,37]. Molecularly, the infiltrative shift is characterized by the promotion of epithelial to mesenchymal transition (EMT) with over-expression of TEM7 (tumor endothelial marker 7), also known as PLXDC1 (plexin domain-containing 1) gene [38]. In addition, EMT is one of the major contributors to therapy failure because of increased invasivity and increased stem-like behavior. We therefore wondered if regorafenib could analogously trigger EMT. To address this point, we exposed U87 and A172 to 7.5 µM regorafenib for 24 or 48 h and measured, by real-time PCR, gene-expression changes of a pool of genes involved in EMT (SNAI1 and SNAI2, TWIST, ID1 and ID3, SERPINE1, and VEGFA). In particular, we focused on TEM7 for its characterized role in bevacizumab-induced infiltrative growth of GBM [38] and on SNAI gene family due to the role of SNAI1 gene in modulating EMT upon regorafenib treatment of hepatocellular carcinoma cells [39].

**Figure 5:**
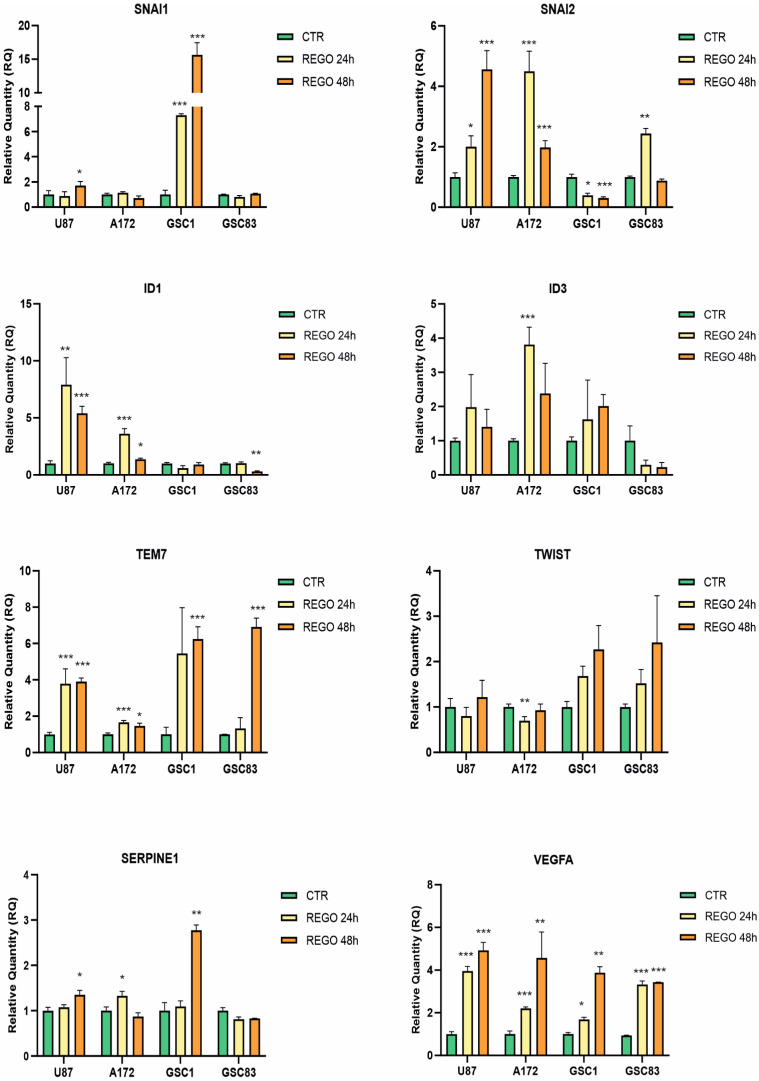
Gene-expression changes upon regorafenib treatment. Real-time PCR analyses of a pool of EMT-related genes, performed on both GBM cell lines and patient-derived GSCs. Cells were treated with 7.5 µM regorafenib for 24 and 48 h. Relative quantities were calculated normalizing for TBP and are given relative to control (vehicle-treated) cells. *n* = 3 biological replicates. * *p* value < 0.05; ** *p* value < 0.01; *** *p* value < 0.001.

Interestingly, TEM7, VEGFA, ID1 and SNAI2, were over-expressed in U87 and A172, at both 24 and 48 h of treatment. The regulation of the other genes examined was more variable and cell-line-specific (Figure 5).

We then asked whether the involvement of the EMT pathway observed in GBM established cell lines also characterizes GSCs. Figure 5 shows the results of gene-expression experiments performed on GSC#1 and GSC#83 exposed to 7.5 µM regorafenib for 24 or 48 h. In the GSC model, the gene-expression regulation appears to be more cell strain-specific than in GBM established cell lines. SNAI1 was significantly up-regulated in GSC#1, while we did not detect any significant regulation of SNAI1 in GSC#83. SnaI2 was significantly down-regulated in GSC#1 following either 24 or 48 h of regorafenib exposure, while it was up-regulated in GSC#83 at 24 h treatment. Remarkably, and coherently with what was observed in U87 and A172, TEM7 and VEGFA were up-regulated by regorafenib in both GSC#1 and GSC#83, although with a slower kinetics when compared to U87 and A172.

### 3.4. Characterization of U87, A172, GSC#1, and GSC#83 3D Spheroids to Regorafenib

**Figure 6:**
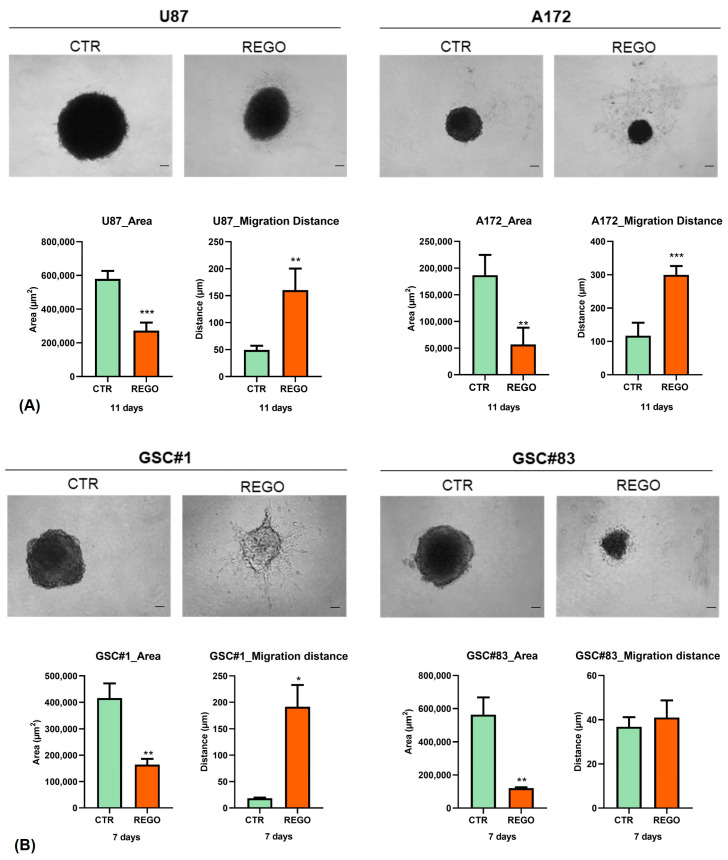
Regorafenib effect on 3D spheroid cultures. Regorafenib, besides limiting spheroid growth, has a pronounced effect on the invading phenotype, visible as the induction of thin branches at spheroid edges ((**A**,**B**), upper panels). Spheroid area is negatively affected by regorafenib. Migration distance, which is a measure of the invading ability of tumor cells, is increased by regorafenib ((**A**,**B**), lower panels). *n* = 5 biological replicates. * *p* value < 0.05; ** *p* value < 0.01; *** *p* value < 0.001. Magnification 4×, scale bar 100 µm.

In comparison with 2D cell-culture models, 3D spheroids are able to accurately mimic some features of solid tumors, such as their spatial architecture, physiological responses, secretion of soluble mediators, gene-expression patterns, and drug resistance mechanism [40,41]. In order to extend the in vitro characterization of GBM tumor cells’ response to regorafenib, we established 3D spheroids from U87, A172, GSC#1, and GSC#83. Cells were seeded in 96-well ultra-low attachment (ULA) plates and spheroid formation was induced by plating cells in 20% methylcellulose in culture medium. We allowed spheroids to grow for 48 h before starting regorafenib treatment. Figure 6A shows bright-field microscopy examination of 3D spheroids. Overall, regorafenib significantly impaired tumor spheroid growth in all the cell models tested, as addressed by measuring spheroid diameters and area of control (vehicle-treated) vs. regorafenib-treated spheroids (Figure 6B). Strikingly, starting from three days of treatment, we observed the presence of invading protrusions in treated spheroids deriving from U87, A172, and GSC#1. Conversely, GSC#83-derived spheroids did not exhibit a significant increase in invading ability, as addressed by measuring the migration distance of cells protruding from the spheroid (Figure 6B, migration distance graphs). Invading cells migrated over time from the edge of the spheroids, invading the surrounding matrix. We observed the same invading phenotype upon regorafenib administration in spheroids cultured in a collagen matrix, which was characterized by a higher stiffness with respect to methylcellulose (not shown).

**Figure 7:**
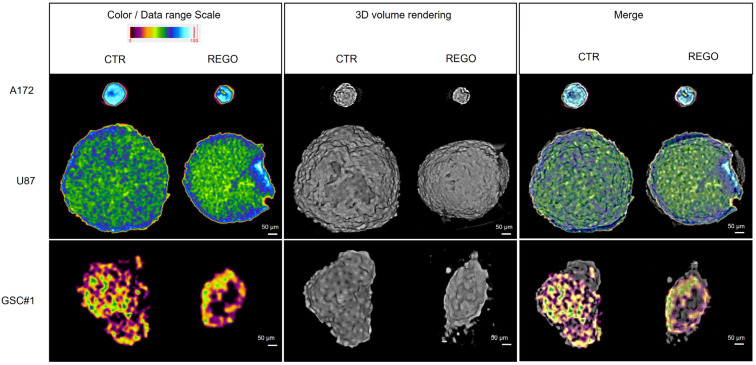
MicroCT analysis of 3D tumor spheroids. Left panels show the sectioning of spheroids in the central transverse plane. Samples were treated with a contrast agent and the color/data range scale indicates the difference in density related to the contrast absorption rate. Middle panels show the three-dimensional images of spheroids, highlighting, in particular, the spheroids’ outer surface. All regorafenib-treated spheroids are modified in shape and angularity compared to control spheroids. Scale bar: 50 µM; resolution of 4.8 µM voxel/size.

To further characterize tumor spheroids, we analyzed them by X-ray microtomography (microCT). This technique allows the fine analysis of spheroid volumes, being a bioimaging system which develops a three-dimensional virtual model of the sample in a non-destructive way and with a micrometric spatial resolution. Tumor spheroids were established in 20% methylcellulose or collagen and exposed to 7.5 µM regorafenib for seven days before fixation and microCT analysis (see Section 2 for experimental procedure details). Consistent with the Image J analyses of microscopy acquisitions (Figure 7), we observed a significant reduction in the dimensions of all spheroids upon regorafenib treatment, as shown by the three-dimensional images of the spheroids shown in Figure 7 and in Supplementary Videos. Unfortunately, we could not analyze GSC#83 spheroids by MicroCT since the handling procedure was not compatible with GSC#83 spheroids stiffness and resulted in the disassembly of the tridimensional structure of the spheroid. Overall, MicroCT analysis confirmed that regorafenib negatively affects growth and modifies spheroid shape, eventually resulting in less defined and less sharp edges than control spheroids. Finally, as a technical note, it is important to highlight that for MicroCT analysis the use of collagen matrix is more suitable than methylcellulose since it confers higher spheroids stability and does not alter the drug-induced changes in spheroid shape.

In summary, the observations obtained in 3D spheroids further support the idea that regorafenib, besides limiting tumor cells’ growth, might induce an invading phenotype, similarly to that already described in GBM treated with bevacizumab [35,38].

**Figure 8:**
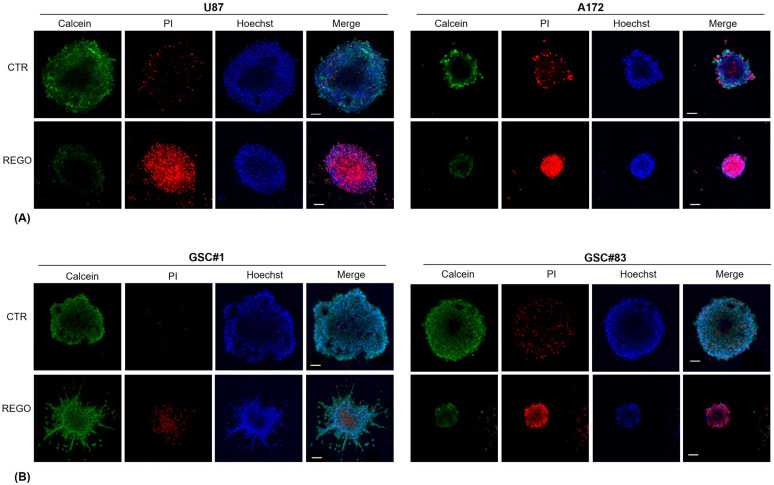
Combined fluorescence images of 3D tumor U87 and A172 (**A**) and GSCs (**B**) spheroids. Tumor spheroids were stained with calcein, propidium and Hoechst, for staining metabolically active cells, dead cells, and cell nuclei, respectively, and analyzed by confocal microscope. Magnification 10×, scale bar 100 µm.

To further characterize regorafenib-treated tumor spheroids, we stained them with calcein AM, propidium and Hoechst [42]. In more detail, cell spheroids were treated with regorafenib for 11 days before staining with calcein AM (to measure metabolically active cells), with propidium iodide (to mark dead cells with a compromised membrane), and with Hoechst (to stain all nucleated cells). As shown in Figure 8, U87- and A172-derived control (vehicle-treated) spheroids exhibited calcein AM-positive cells mainly localized in the outer layers of the 3D structure, where cells are in a more favorable environment, exposed to nutrients and oxygen. Only a weak propidium-positivity was found, with stained cells localizing in the spheroid core, suggesting that only a low percentage of the tumor cells composing the spheroids were necrotic. Conversely, in regorafenib-treated spheroids, we observed a strong increase in propidium fluorescence, which appears in all spheroid compartments, and a strong decrease of calcein-positive cells.

Overall, data obtained in the 3D GBM tumor and cancer stem cell models confirm the anti-tumor effect of regorafenib addressed in the 2D cell cultures in terms of inhibition of tumor cell growth. Notably, cells migratory ability was enhanced by regorafenib.

## 4. Discussion

Regorafenib is a multikinase inhibitor targeting a variety of receptors, such as VEGFR1-3, TIE2, KIT, RET, RAF1, BRAF, PDGFR, and FGFR. It is approved as monotherapy for the treatment of hepatocellular carcinoma, gastrointestinal stromal tumors, and colorectal cancers [43,44,45]. In 2019, the phase II randomized multicenter trial REGOMA provided encouraging results about clinical effectiveness of regorafenib on relapsed GBM patients in comparison with lomustine. The study pointed out a significant increase of the OS of regorafenib-treated patients. Neuro-radiological assessment (according to RANO criteria) and PFS also benefitted from regorafenib [9]. The results of the REGOMA trial are impressive since no significant improvement in GBM therapeutic approach has been achieved in the last 25 years. Consequently, regorafenib was included in the NCCN 2020 guidelines as a preferred regimen for recurrent GBM patients, and also the Italian Agency of Medicine (AIFA) approved its use for Italian patients in October 2019. Very recently, a retrospective clinical study was published, where authors reported the activity, efficacy, and safety analysis of regorafenib treatment in patients with relapsed GBM who had received chemoradiation therapy as first-line treatment. Again, regorafenib proved to have an effectiveness comparable to that of REGOMA, with an even longer survival time [10]. Although clinical data are accumulating, demonstrating encouraging efficacy and tolerability of regorafenib in GBM patients [10,11,15,46], the mechanisms governing GBM response to regorafenib have been poorly investigated. Santangelo and colleagues [12] identified five biomarkers which could help in identifying a subpopulation of GBM patients exhibiting a striking survival advantage when treated with regorafenib. Analogously, Chiesa et al. [47] very recently published the results of an NGS study performed on a cohort of 30 GBM patients harboring recurrent glioblastoma and treated with regorafenib. They identified two different mutations which seem to be associated with a poor response to regorafenib: a mutation in the EGFR pathway and a mutation in the mitogen-activated protein-kinase (MAPK) pathway. Our work fits in this frame and its aim is to provide an in vitro characterization of GBM cell lines and of patient-derived GSCs exposed to regorafenib. Although we did not go through the mechanisms underlying GBM cells’ response to regorafenib, we pinpointed some relevant aspects which deserve future investigation. Basically, the message emerging from the present study is that regorafenib limits GBM cell proliferation in all the cellular models tested, both in 2D cell cultures and in 3D spheroids. Apoptosis is not a key mechanism through which regorafenib induces cell death in GBM cells (as measured by annexin V/propidium iodide staining), although we detected a modest increase in apoptotic cells upon regorafenib treatment. Autophagy might represent the principal mechanism, as previously published [14]. Regorafenib may also have a cytostatic effect which is compatible with the increased proportion of U87 and A172 cells in G1 phase. Interestingly, regorafenib administration is accompanied by regulation of genes involved in EMT. The trend which emerges from our limited survey of regorafenib-regulated genes is that all cell lines showed up-regulation of VEGF and TEM7 mRNAs. The first one encodes for a growth factor which is a major regulator of angiogenesis and adaptation to hypoxia, the second encodes for an endothelial cell marker associated with the infiltrative growth of GBM tumor promoted by the anti-VEGF monoclonal antibody bevacizumab [38]. Our data on regorafenib-dependent induction of EMT-driving transcription factors are more heterogeneous among the four cell lines used. This finding should not come as a surprise due to the heterogeneity of GBM and GCS cell lines which mirrors the heterogeneity of human GBM [16,28]. Additionally, EMT relies on a complex network of redundant transcription factors. In our case a consistent picture emerges. In all the cell lines tested, regorafenib induced up-regulation of at least one member of SNAI family (SNAI1 in GCS#1 and possibly in U87; SNAI2 in U87, A172, and GSC-83) and, with the possible exception of A172, of a member of the ID family (ID-1 in U87 and A172; ID3 in U87, A172 and GCS#1). Regarding Twist, its induction appears to be confined to GSCs. It should be considered, in any event, that our analysis was restricted to the time points of 24 and 48 h. An analysis focused on earlier times may be more informative since transcription-factor regulation generally represents an early, often transient, response to cell perturbations. Consistent with the up-regulation of VEGF and TEM7 is the increased migration ability, documented in tumor spheroids as the presence of irregular spheroids edges, with extended thin branches invading the surrounding matrix. EMT gene regulation was previously described in vitro in hepatocellular carcinoma cells (HCCs) exposed to regorafenib [39]. As opposed to what we find in GBM cells, regorafenib inhibits migration and invasion in HCC targeting ID1-mediated EMT. Strikingly, the pro-migratory phenotype acquired by GBM cells upon regorafenib treatment resembles what was already observed, both in in vitro systems and in vivo, following bevacizumab treatment. Bevacizumab received accelerated approval from the US Food and Drug Administration for treatment of recurrent GBM in the United States, based on the success of two phase II clinical trials [48,49]. Unfortunately, bevacizumab disappointed the expectations since it did not prove to have a positive effect on OS. A sustained response to bevacizumab therapy is not the rule in GBM, whereas tumor regrowth after initial response is frequently seen. In addition, treatment with bevacizumab may trigger a phenotypic change in GBM that acquires a gliomatosis-like growth pattern [35,37]. The infiltrative growth of GBM after bevacizumab treatment was described in several studies on both a clinical [50,51] and a pathological basis [35,52,53] and could represent a limitation of its therapeutic effectiveness. Our in vitro data demonstrate an antitumorigenic effect of regorafenib on GBM growth, which reflects the clinical effectiveness. At the same time, we point out the induction of an invading phenotype resembling the one elicited by bevacizumab. GSC#83 seems to be more sensitive to regorafenib than GSC#1, both in terms of growth inhibition (see Figure 6) and because GSC#83 does not respond to regorafenib with an enhanced migratory ability. It is interesting to note, as a mere speculation, that GSC#83 are EGFRvIII-positive as opposed to GSC#1. In their recent paper, Chiesa and colleagues [47] identified EGFR pathway mutation as a characteristic associated with poor response to regorafenib in GBM patients.

The use of anti-angiogenic drugs, and the following inhibition of angiogenesis, protects the blood–brain barrier (BBB), which is less disrupted, eventually resulting in a decrease of tumor enhancement even in rapidly growing tumors [54]. This mechanism is at the basis of “pseudoresponse”, which describes the decrease in tumor enhancement according to MacDonald’s criteria, with no significant antitumor effect [55,56]. Of note, a recent paper reported about an MRI pattern of pseudoprogression in a case of recurrent GBM treated with regorafenib. The authors observed an MRI pattern partially overlapping with those largely described for GBM patients treated with bevacizumab at tumor recurrence [57]. In this case report, authors describe a T2-dominant growth pattern, despite initial decrease in tumor-enhancing T1-weighted images. They detected an infiltrative non-enhancing relapse, accompanied by progressive clinical decline preceding by about 90 days the detection of radiological disease progression as addressed by McDonald criteria and Response Evaluation Criteria in Solid Tumors (RECST). The issue of regorafenib-dependent pseudoprogression in GBM patients has been poorly described [6] and, to the best of our knowledge, our data are the first to suggest the idea that regorafenib might elicit a pro-invasive phenotype in GBM.

## 5. Conclusions

Although our data require confirmation by independent studies, they set a word of caution in regorafenib clinical use and strongly support the need for studying the molecular mechanisms involved in regorafenib response of GBM cells.

## Figures and Tables

**Table 1:** Real-time PCR primer sequences.

ID Gene	Primer Forward	Primer Reverse
TBP	TGCCCGAAACGCCGAATATAATC	TGGTTCGTGGCTCTCTTATCCTC
VEGFA	CCTTGCTGCTCTACCTCCAC	CAACTTCGTGATGATTCTGC
Id1	TGAACGGCTGTTACTCACG	CAACTGAAGGTCCCTGATG
Id3	GAGAGGCACTCAGCTTAGCC	TCCTTTTGTCGTTGGAGATGAC
SERPINE1	GCAAGGCACCTCTGAGAACT	TCACCAAAGACAAGGGCCAG
SnaI1	AAGCCTAACTACAGCGAGCT	GAGTCCCAGATGAGCATTGG
SnaI2	AGCATTTCAACGCCTCCAAA	TGGTTGTGGTATGACAGGCA
TEM7	GTCAAAACCGGCCTATCGGA	GATGCTCCTTCGCCGAGATT
TWIST	AGTCTTACGAGGAGCTGCAG	ATCTTGCTCAGCTTGTCCGA

**Table 2:** Clinical features of GBM patients originating GSC lines.

GSC#	Age/Sex	Tumor Location	Histology (WHO Grade)	Molecular Profile	PFS */OS (mos)
1	40/M	Right frontal	Glioblastoma (IV)	MGMT M, IDH wt, EGFRvIII neg, VEGF hyper	6.5/12.5
61	59/M	Occipital	Glioblastoma (IV)	MGMT UM, IDH wt, EGFRvIII pos, VEGF normal	3/6
83	52/M	Temporal	Glioblastoma (IV)	MGMT UM, IDH wt, EGFRvIII pos, VEGF hyper	3/8
366	54/M	Temporal	Glioblastoma (IV)	MGMT UM, IDH wt, EGFRvIII neg, VEGF hyper	42/60.5
450	76/M	Temporal	Glioblastoma (IV)	MGMT M, IDH wt, EGFRvIII neg, VEGF hyper	3/6
493	49/F	Parietal	Glioblastoma (IV)	MGMT M, IDH wt, EGFRvIII pos, VEGF hyper	11/22.5

*, PFS refers to the standard chemoradiation with temozolomide.

**Table 3:** Regorafenib IC50 values.

Cell Line	IC50 (μM)
A172	2.4
U87	6.3
GSC#1	4.7
GSC#61	6.2
GSC#83	5.4
GSC#450	3.3
GSC#366	6.2
GSC#439	3.5

## Data Availability

Not applicable.

## Supplementary Materials

The following supporting information can be downloaded at: https://www.mdpi.com/article/10.3390/cancers14246193/s1, Video S1: MicroCT analysis video. Figure S1: FACS profiles of propidium iodide-labeled cells, Figure S2: Clonogenesis assay on GSC cells. Regorafenib impairs GSC ability to develop spheres, Figure S3: Annexin staining of cells treated with regorafenib for 24 h, Figure S4: Original western blot.
