# Supplementary material for: Characterization of Glioblastoma Cells Response to Regorafenib
Source: Cancers (Basel). 2022 Dec 15;14(24):6193. doi: 10.3390/cancers14246193 (PMC9777191; doi:10.3390/cancers14246193)
Supplement: Supplementary file 1 [file cancers-14-06193-s001.zip › cancers-2071996-supplementary/cancers-2071996 supplementary figure.pdf]

cancers MDPI

# Supplementary Material: Characterization of Glioblastoma Cells Response to Regorafenib

**Maria Patrizia Mongiardi, Mariachiara Buccarelli, Alessia Formato, Elisa Orecchini, Maria Salbini, Valentina Ricci, Tiziana Orsini, Sabrina Putti, Silvia Chiesa, Lucia Ricci-Vitiani, Quintino Giorgio D'Alessandris, Roberto Pallini, Andrea Levi and Maria Laura Falchetti**

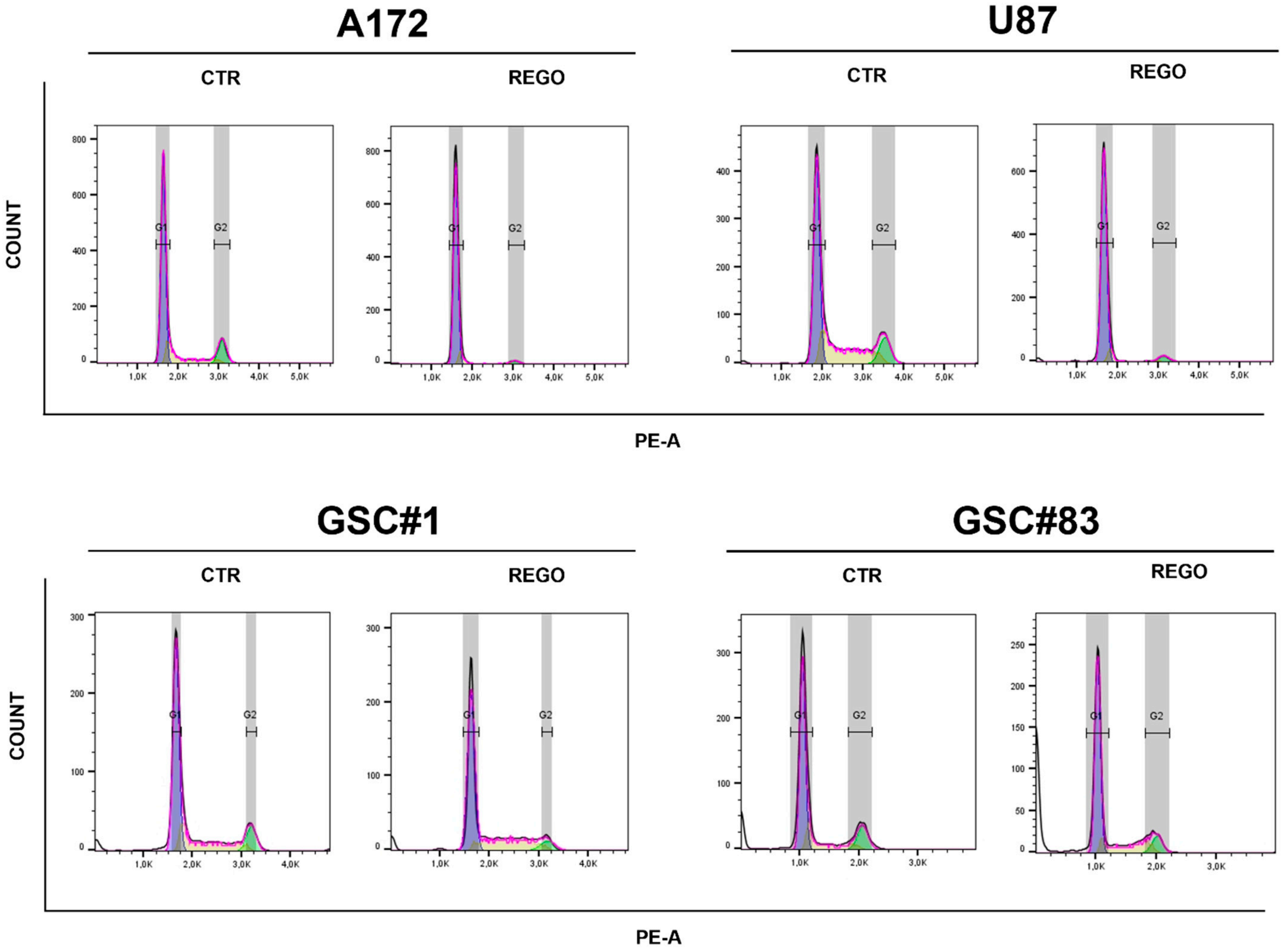


**Supplementary Figure S1.** FACS profiles of propidium iodide-labeled cells.

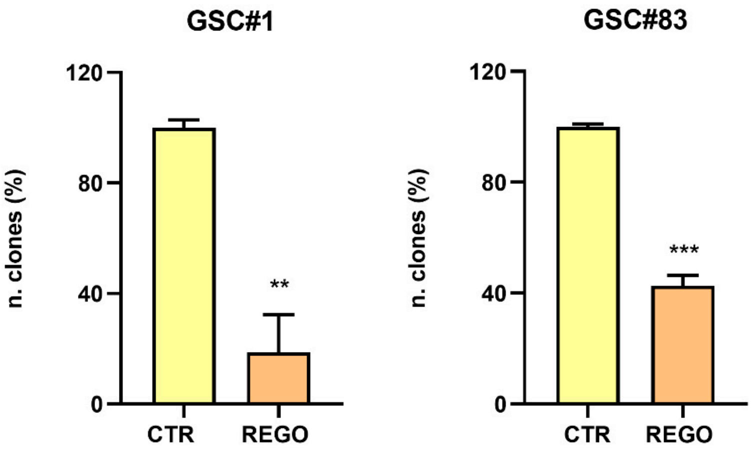


**Supplementary Figure S2.** Clonogenesis assay on GSC cells. Regorafenib impairs GSC ability to develop spheres. ** *p* value < 0.01; *** *p* value < 0.001.

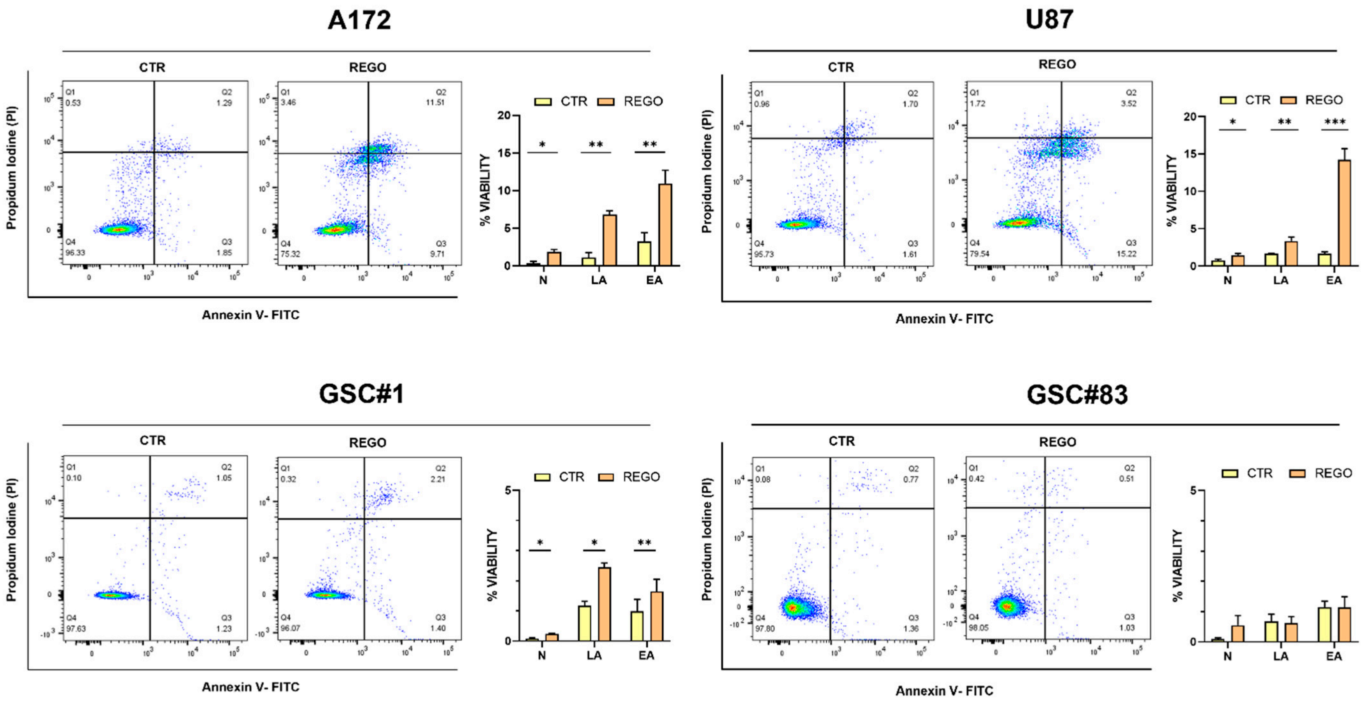


**Supplementary Figure S3.** Annexin staining of cells treated with regorafenib for 24 hours. Cells were double stained by An-nexin V and by Propidium iodide, for apoptotic and dead cells labeling, respectively. N: necrotic cells; LA: late apoptotis; EA: early apoptosis. * *p* value < 0.05; ** *p* value < 0.01; *** *p* value < 0.001.

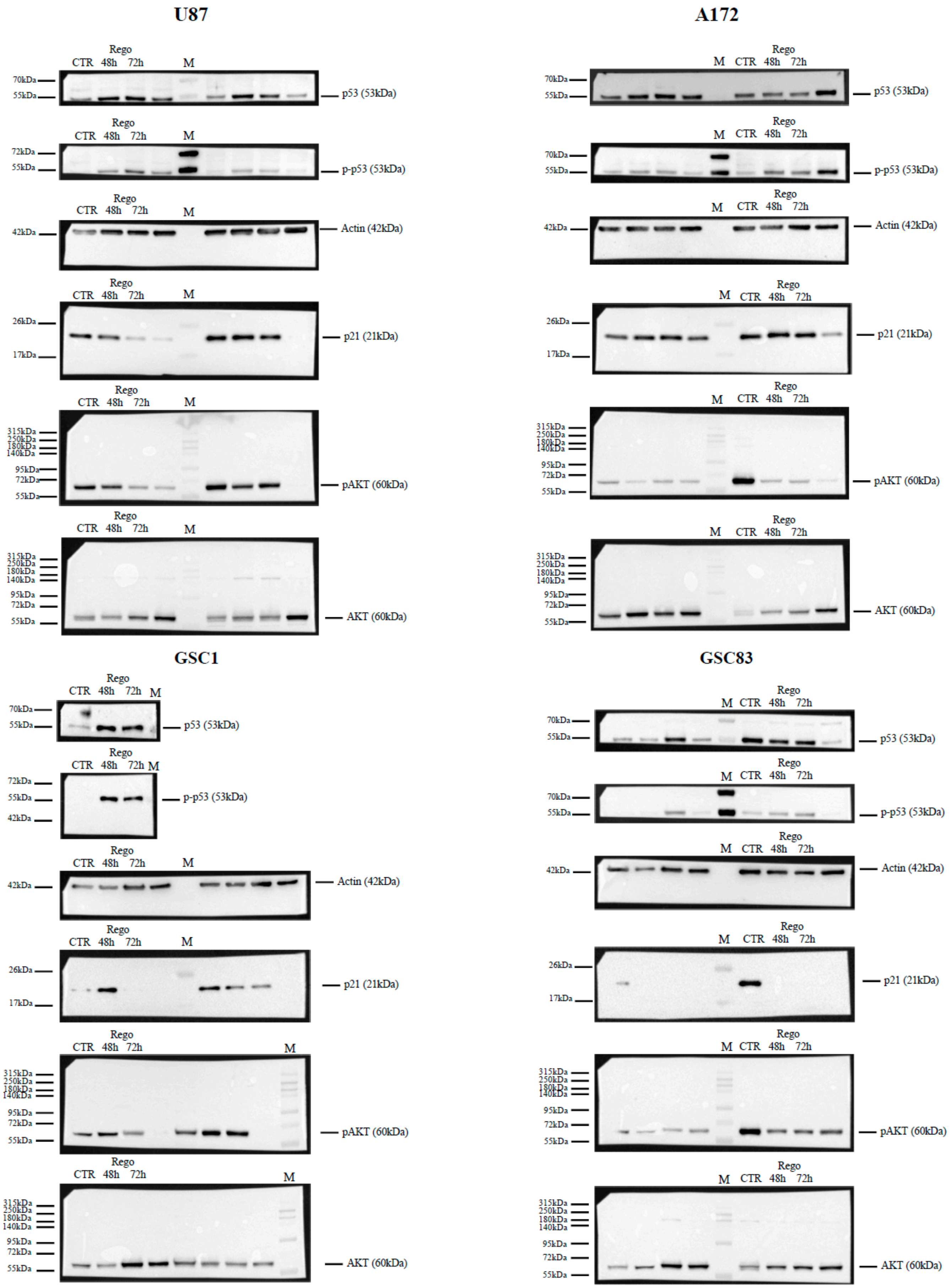
U87
A172
GSC1
GSC83
Rego
CTR 48h 72h
M
p53 (53kDa)
p-p53 (53kDa)
Actin (42kDa)
p21 (21kDa)
pAKT (60kDa)
AKT (60kDa)

| | **U87** |
|---|---|
| | |
| | **P-P53** |
| CTR 48h | 1 |
| REGO 48h | 11,05 |
| REGO 72h | 25,05 |
| | |
| | **P53** |
| CTR 48h | 1 |
| REGO 48h | 2,89 |
| REGO 72h | 3,12 |
| | |
| | **P-P53/P53 TOT** |
| CTR 48h | 1 |
| REGO 48h | 3,82 |
| REGO 72h | 8,03 |
| | |
| | **P21** |
| CTR 48h | 1 |
| REGO 48h | 0,8 |
| REGO 72h | 0,21 |
| | |
| | **ACTIN** |
| CTR 48h | 1 |
| REGO 48h | 1,14 |
| REGO 72h | 1,09 |
| | |
| | **P21/ACTIN** |
| CTR 48h | 1 |
| REGO 48h | 0,70 |
| REGO 72h | 0,19 |
| | |
| | **P-AKT** |
| CTR 48h | 1 |
| REGO 48h | 0,75 |
| REGO 72h | 0,28 |
| | |
| | **AKT** |
| CTR 48h | 1 |
| REGO 48h | 0,84 |
| REGO 72h | 1,33 |
| | |
| | **P-AK/AKT TOT** |
| CTR 48h | 1 |
| REGO 48h | 0,89 |
| REGO 72h | 0,21 |

| | **A172** |
|---|---|
| | |
| | **P-P53** |
| CTR 48h | 1 |
| REGO 48h | 2,48 |
| REGO 72h | 2,31 |
| | |
| | **P53** |
| CTR 48h | 1 |
| REGO 48h | 0,9 |
| REGO 72h | 0,8 |
| | |
| | **P-P53/P53 TOT** |
| CTR 48h | 1 |
| REGO 48h | 2,76 |
| REGO 72h | 2,89 |
| | |
| | **P21** |
| CTR 48h | 1 |
| REGO 48h | 1,14 |
| REGO 72h | 0,98 |
| | |
| | **ACTIN** |
| CTR 48h | 1 |
| REGO 48h | 0,77 |
| REGO 72h | 0,74 |
| | |
| | **P21/ACTIN** |
| CTR 48h | 1 |
| REGO 48h | 1,48 |
| REGO 72h | 1,32 |
| | |
| | **P-AKT** |
| CTR 48h | 1 |
| REGO 48h | 0,09 |
| REGO 72h | 0,09 |
| | |
| | **AKT** |
| CTR 48h | 1 |
| REGO 48h | 2,68 |
| REGO 72h | 3,9 |
| | |
| | **P-AK/AKT TOT** |
| CTR 48h | 1 |
| REGO 48h | 0,03 |
| REGO 72h | 0,02 |

| | **GSC1** |
|---|---|
| | |
| | **P-P53** |
| CTR 48h | 1 |
| REGO 48h | 126,97 |
| REGO 72h | 91,78 |
| | |
| | **P53** |
| CTR 48h | 1 |
| REGO 48h | 4,68 |
| REGO 72h | 4,6 |
| | |
| | **P-P53/P53 TOT** |
| CTR 48h | 1 |
| REGO 48h | 27,13 |
| REGO 72h | 19,95 |
| | |
| | **P21** |
| CTR 48h | 1 |
| REGO 48h | 3,47 |
| REGO 72h | 0,22 |
| | |
| | **ACTIN** |
| CTR 48h | 1 |
| REGO 48h | 1 |
| REGO 72h | 1,87 |
| | |
| | **P21/ACTIN** |
| CTR 48h | 1 |
| REGO 48h | 3,47 |
| REGO 72h | 0,12 |
| | |
| | **P-AKT** |
| CTR 48h | 1 |
| REGO 48h | 1,47 |
| REGO 72h | 0,96 |
| | |
| | **AKT TOT** |
| CTR 48h | 1 |
| REGO 48h | 0,78 |
| REGO 72h | 3,23 |
| | |
| | **P-AKT/AKT TOT** |
| CTR 48h | 1 |
| REGO 48h | 1,88 |
| REGO 72h | 0,30 |

| | **GSC83** |
|---|---|
| | |
| | **P-P53** |
| CTR 48h | 1 |
| REGO 48h | 1,5 |
| REGO 72h | 2,3 |
| | |
| | **P53** |
| CTR 48h | 1 |
| REGO 48h | 0,53 |
| REGO 72h | 0,6 |
| | |
| | **P-P53/P53 TOT** |
| CTR 48h | 1,00 |
| REGO 48h | 2,83 |
| REGO 72h | 3,83 |
| | |
| | **P21** |
| CTR 48h | 1 |
| REGO 48h | 0 |
| REGO 72h | 0 |
| | |
| | **ACTIN** |
| CTR 48h | 1 |
| REGO 48h | 0,78 |
| REGO 72h | 0,71 |
| | |
| | **P21/ACTIN** |
| CTR 48h | 1 |
| REGO 48h | 0,00 |
| REGO 72h | 0,00 |
| | |
| | **P-AKT** |
| CTR 48h | 1 |
| REGO 48h | 0,26 |
| REGO 72h | 0,27 |
| | |
| | **AKT TOT** |
| CTR 48h | 1 |
| REGO 48h | 1,21 |
| REGO 72h | 1,3 |
| | |
| | **P-AKT/AKT TOT** |
| CTR 48h | 1 |
| REGO 48h | 0,21 |
| REGO 72h | 0,21 |

**Supplementary Figure S4.** Original western blot.
